# Mitochondrial dysfunction in Fragile X syndrome and Fragile X-associated tremor/ataxia syndrome: prospect use of antioxidants and mitochondrial nutrients

**DOI:** 10.1007/s11033-024-09415-7

**Published:** 2024-04-05

**Authors:** Giovanni Pagano, Alex Lyakhovich, Federico V. Pallardó, Luca Tiano, Adriana Zatterale, Marco Trifuoggi

**Affiliations:** 1 Department of Chemical Sciences, Federico II Naples University, via Cintia, Naples, I-80126 Italy; 2 Sabanci University, Tuzla, Istanbul, 34956 Turkey; 3 Department of Physiology, Faculty of Medicine and Dentistry, University of Valencia-INCLIVA, CIBERER, Valencia, E-46010 Spain; 4 Department of Life and Environmental Sciences, Polytechnical University of Marche, Ancona, I-60121 Italy; 5 Department of Genetics, ASL Napoli 1, Naples, I-80144 Italy

**Keywords:** Fragile X syndrome (FXS;FXTAS), Mitochondrion, Coenzyme Q10, Carnitine, Alpha-lipoic acid

## Abstract

Fragile X syndrome (FXS) is a genetic disorder characterized by mutation in the FMR1 gene, leading to the absence or reduced levels of fragile X Messenger Ribonucleoprotein 1 (FMRP). This results in neurodevelopmental deficits, including autistic spectrum conditions. On the other hand, Fragile X-associated tremor/ataxia syndrome (FXTAS) is a distinct disorder caused by the premutation in the FMR1 gene. FXTAS is associated with elevated levels of FMR1 mRNA, leading to neurodegenerative manifestations such as tremors and ataxia.

Mounting evidence suggests a link between both syndromes and mitochondrial dysfunction (MDF). In this minireview, we critically examine the intricate relationship between FXS, FXTAS, and MDF, focusing on potential therapeutic avenues to counteract or mitigate their adverse effects. Specifically, we explore the role of mitochondrial cofactors and antioxidants, with a particular emphasis on alpha-lipoic acid (ALA), carnitine (CARN) and Coenzyme Q10 (CoQ10). Findings from this review will contribute to a deeper understanding of these disorders and foster novel therapeutic strategies to enhance patient outcomes.

## Introduction

Fragile X syndrome (FXS) is the most common inherited intellectual and autism spectrum disorder in humans. FXS is due to a CGG trinucleotide expansion repetition that produces decreased expression of the fragile X Messenger Ribonucleoprotein 1 (FMRP) previously reported as fragile X mental retardation protein 1 [1, 2]. This protein potentially regulates a high number of mRNAs involved in neuronal development and neuroplasticity, the brain’s ability to reorganize itself by forming new neural connections throughout life in response to various experiences or environmental changes [3]. This decreased expression of FMRP or the loss of its function induces an imbalance in inhibitory and excitatory neurotransmitters and is associated with intellectual disability and other cognitive impairments. As reported by Zhang et al. [4], FMRP expression is widespread but is especially high in the brain and testis. In the brain, FMRP has been implicated in dendritic spine maturation, synapse formation, and synaptic plasticity, with relevant roles in mRNA transport and translational control, and in messenger ribonucleo-protein (mRNP) complexes that associate with polyribosomes and in mRNA nuclear export. Recently, Geng et al. [5] reported that FMRP interacts with the voltage-dependent anion channel (VDAC) regulating the formation and function of endoplasmic reticulum (ER)-mitochondria contact sites (ERMCSs), structures that are critical for mitochondrial calcium (mito-Ca^2+^) homeostasis. This is important since inhibition of VDAC or other ERMCS components restored synaptic structure, function, and plasticity and rescued locomotion and cognitive functions.

Among the clinical consequences of FXS, mainly involving neurodevelopmental abnormalities, Fragile X-associated tremor/ataxia syndrome (FXTAS) is characterized by motor deficits, impaired energy metabolism in adult patients and mitochondrial dysfunction (MDF) [6–8]. The present minireview focuses on some selected reports on the combination of FXS and FXTAS with MDF and with the role of some synthetic or naturally occurring antioxidants that may result in protective – or adjuvant - effects on the FXS/FXTAS phenotype.

### Oxidative stress and tested antioxidants in FXS and FXTAS

In FXS, oxidative stress (OS) has been implicated as a contributing factor to the neurodegenerative manifestations seen in the disorder. In particular, OS markers as well as oxidative damage in the form of lipid peroxidation and protein carbonylation were found in in FXS mice models [9]. This study also demonstrated impaired antioxidant defense mechanisms in FXS individuals, suggesting an imbalance between reactive oxygen species (ROS) production and antioxidant capacity. In FXTAS, oxidative stress has also been implicated in the disease pathophysiologyy [10]. Several antioxidants or their combinations have been tested in counteracting FXS-/FXTAS-associated damage in both clinical trials and in a number of models, including patient cells and rodents with specific mutations [11, 12]. Treatment with a natural neurosteroid allopregnanolone was able to reduce OS and improved mitochondrial function in a 12-week open-label intervention study of six males with FXTAS [13]. Table 1 summarizes several antioxidants or their combinations that have been tested in counteracting FXS/FXTAS-associated damage in several models, as well as in clinical trials [15, 18–23]. The current and prospect utilization of antioxidants in the treatment of FXS/FXTAS has been presented in further reports and reviews, altogether raising the realistic prospects of adjuvant therapies as tools to ameliorate the health status and life expectancy of FXS/FXTAS patients [24–29].

**Table 1:** Use of selected antioxidants in inducing adjuvant effects in FXS/FXAS models

Agent	Model	Effects	Dose	Ref.
Allopregnanolone	pilot study on FXTAS patients	reduced OS and improved mitochondrial	2,4,6 mg	[13]
		functions in FXS	weekly	
Sulforaphane	fibroblasts from	beneficial effect of sulforaphane exerted	5 µM for 72 h	[16]
	FXTAS patients	through NRF2 on brain function,		
		bioenergetics, and antioxidant defense		
Epigallocatechin-3-gallate (EGCG)	*Fmr1* knockout mice;	improved cognition (visual episodic memory)	10 mg/kg	[22]
	phase I clinical trial	and functional competence	5–7 mg/kg/day	
Melatonin	clinical trials	protective effects	3 mg/day	[17–19]
Ascorbic acid and α-tocopherol	FXTAS patients	reversed pathophysiological hallmarks	10 mg/kg/day	[21]
		(free radical overproduction, oxidative stress,		
		Rac1 and α-PKC activation), macroorchidism,		
		and behavior and learning deficits		

### Mitochondrial dysfunction in FXS and FXTAS

Mitochondrial dysfunction in FXS and FXTAS has been increasingly recognized as a contributing factor to the pathogenesis of these disorders. Abnormalities in the transport of nuclear-encoded proteins into mitochondria have been observed in carriers with or without FXTAS, suggesting a potential role of mitochondrial dysfunction in the pathophysiology [7, 8]. In FXS, deficiency of FMRP has been linked to impaired mitochondrial function, altered mitochondrial morphology, and disrupted calcium homeostasis [7]. Similarly, FXTAS has been associated with mitochondrial abnormalities, including decreased activities of respiratory chain complexes in cells derived from FXTAS patients [8].

Studies in mouse models of FXS have revealed elevated levels of reactive oxygen species (ROS), lipid and protein oxidation, implicating mitochondrial enzymes involved in oxidative homeostasis, particularly in wild-type and *Fmr1* knockout glial cells [24]. Moreover, Fmr1 deletion in astrocytes has been shown to result in decreased mitochondrial respiration and increased ROS production [25, 26]. Interestingly, the impact of the premutation, as observed in the FMR1 gene, extends beyond FXS and has been implicated in other neurodegenerative diseases, such as Parkinson’s disease (PD) [27]. Notably, some cases of PD bear striking resemblance to FXTAS [29].

Considering recent studies suggesting potential treatments for PD involving a combination of CoQ, B-vitamins/NADH, CARN, vitamin D, and ALA [28], it is plausible to explore the therapeutic potential of these mitochondrial nutrients (MNs) for FXS and FXTAS.

### Mitochondrial cofactors in FXS and FXTAS

The involvement of some factors in FXS and FXTAS with prooxidant effects may be counteracted by some antioxidants from natural or synthetic sources, pointing to the beneficial roles of MNs [29–32]. The distinct, yet complementary roles of antioxidants and of MNs are summarized in Table 2. A few studies have reported on the protective roles of CARN alone or in combination with antioxidants [33–35]. It should be mentioned that an analytical review of CARN-focused clinical trials failed to confirm a protective role of CARN in FXS patients [36].

The role of CoQ10 in FXS (Fmr1 knockout) mutant mice has been reported in two studies [37, 38], where altered CoQ10 content, with tissue-specific differences in forebrain and heart mitochondria in a newborn mouse model of FXS (Fmr1 knockout), was linked to MDF and cellular dysfunction. In particular, altered CoQ10 biosynthesis seems to affect mitochondria permeability transition pore activity and thermogenic control in brown adipose tissues. These findings further stress the link between FMRP and CoQ10 biosynthesis that could play a role in altered neurodevelopment [38].

No direct information, to the best of our knowledge, is available on the role(s) of another key MN, ALA, endowed to be an essential nutrient for mitochondrial functions, namely in the TCA (Krebs) cycle and as a recognized antioxidant [39]. Several reports on ALA-associated protective roles of ALA in other pathologies [40–42]. Further research is expected on this subject.

**Table 2:** Involvement of mitochondrial cofactors (mitochondrial nutrients, MNs) in FXS/FXTAS cells

Mitochondrial cofactor	Model	Effects	Dose	Ref.
Acetyl-carnitine (CARN)				
CARN + butyrate	lymphocytes of FXS patients	inhibition of fra(X)(q27.3) expression	1.4–3.2 mM	[33]
CARN	17 FXS patients	modulating energy production; remodelling	50 mg/kg/day	[34]
		production of polyunsaturated fatty acids		
CARN	63 FXS patients	beneficial effect on hyperactive-impulsive behavior	50 mg/kg/day	[35]
Coenzyme Q10 (CoQ10)				
CoQ10	integrity of forebrain mitochondria	CoQ deficiency and an open cyclosporine-sensitive		[37]
	in Fmr1 knockout (KO) mice	channel. Repletion of mitochondrial CoQ within		
	forebrain closed the channel	the Fmr1 KO mice		
CoQ10	leak in Fmr1 KO forebrain mitochondria	CoQ deficiency within BAT mitochondria resulted		[38]
		in abnormal substrate oxidation		
Alpha-lipoic acid (ALA)				
TCA cycle	mitochondrial deficits	anaplerosis of TCA in serine biosynthesis		[42]

## Limitations and conclusion remarks

Limitations of the studies reviewed in this paper include small sample sizes, heterogeneous patient populations, and variability in treatment protocols. Many studies have been conducted using cell lines or mouse models [19, 22, 23], and clinical trials with human subjects have been limited [15, 18, 34–36]. Although some works did not have control groups, making it difficult to draw definitive conclusions about treatment effects, other studies emphasize the importance of conducting clinical trials to assess the potential benefits of MNs and antioxidants in the treatment of FXS/FXTAS [14–24].

Notwithstanding the current limitations on the roles – if any – of MNs, it is worth recalling the recognized prevalence of MDF, along with a prooxidant state, in FXS/FXTAS [2, 7, 11–19, 40–47].

Consistent with the inclusion of FXS/FXTAS among mitochondrial cytopathies, FXS/FXTAS can be subjected to in vitro and ex vivo studies that should determine the optimal dosages and combinations of these treatments, as well as their long-term safety and efficacy. Moreover, it should be noted that MDF and oxidative stress levels may vary among individuals. Therefore, a personalized medicine approach that considers individual variability in these factors may be necessary to optimize response to treatment and minimize potential side effects. Biomarkers such as mitochondrial DNA content, mitochondrial respiration rate, and markers of oxidative stress may be useful in assessing treatment response and guiding the dosage and duration of therapy. Such an approach can provide effective FXS/FXTAS adjuvant treatment.

## Data Availability

No datasets were generated or analyzed during the current study.
